# The Pre-Discharge Oxygen Uptake Efficiency Slope Predicts One-Year Cardiovascular Events in Acute Decompensated Heart Failure Patients

**DOI:** 10.3390/life12091449

**Published:** 2022-09-19

**Authors:** I-Ching Huang, Yi-Jen Chen, Chia-Hsin Chen, Wei-Chun Huang, Ko-Long Lin

**Affiliations:** 1 Department of Physical Medicine and Rehabilitation, Kaohsiung Medical University Hospital, Kaohsiung Medical University, Kaohsiung 80756, Taiwan; 2 Department of Physical Medicine and Rehabilitation, Kaohsiung Municipal Siaogang Hospital, Kaohsiung Medical University, Kaohsiung 81267, Taiwan; 3 Department of Physical Medicine and Rehabilitation, School of Medicine, College of Medicine, Kaohsiung Medical University, Kaohsiung 80756, Taiwan; 4 Department of Physical Medicine and Rehabilitation, Ditmanson Medical Foundation Chia-Yi Christian Hospital, Chia-Yi 60002, Taiwan; 5 Critical Care Center, Cardiovascular Medical Center, Kaohsiung Veterans General Hospital, Kaohsiung 81362, Taiwan; 6 School of Medicine, National Yang-Ming University, Taipei 11221, Taiwan; 7 Department of Physical Medicine and Rehabilitation, Kaohsiung Veteran General Hospital, Kaohsiung 81362, Taiwan; 8 School of Medicine, College of Medicine, National Yang Ming Chiao Tung University, Hsinchu 11221, Taiwan

**Keywords:** heart failure, major adverse cardiovascular event, cardiopulmonary exercise testing, oxygen uptake efficiency slope

## Abstract

(1) Background: Heart failure is a complex disease leading to functional disability. Cardiopulmonary exercise testing (CPET) is the gold standard in assessing aerobic capacity and formulating function-based prognostic stratification; however, patients with acute heart failure after medical treatment usually remain with markedly reduced exercise capacity, leading to early termination of CPET with submaximal testing results. The current study aimed to assess the cardiorespiratory fitness and characteristics of CPET variables of patients after acute heart failure treatment and determine potential CPET variables with prognostic value. (2) Methods: We recruited patients during hospitalization after management of acute heart failure, and pre-discharge CPET was performed. All enrolled patients were followed for one year for major adverse cardiovascular events (MACE). (3) Results: 85 patients were enrolled, with average left ventricular ejection fraction of 30.52%, and peak oxygen consumption of 10.85 mL/min/kg at baseline. The one-year MACE was 50%. Oxygen uptake efficiency slope (OUES) was a significant event predictor, with lower one-year MACE in those with OUES ≥ 1.25 (*p* < 0.001). Cox regression analysis showed a 5.421-fold increased risk of MACE in those with OUES < 1.25 (*p* = 0.004). (4) Conclusions: The current results suggested OUES is a significant prognostic indicator in patients with acute heart failure. This also emphasized the critical role of CPET in patients with heart failure for prognostic stratification.

## 1. Introduction

Heart failure (HF) is a complex disease with various clinical symptoms that lead to functional disability. It is a global pandemic, since it affects around 26 million people world-wide [1]. The prevalence of heart failure is increasing, but the survival rate has improved as well owing to advanced medical treatments and diagnostic technology for an aging society [2]. HF can be classified into three subtypes according to left ventricular ejection fraction (LVEF): HF with reduced ejection fraction (HFrEF) (LVEF ≤ 40%), HF with preserved ejection fraction (HFpEF) (LVEF ≥ 50%) and HF with mid-range ejection fraction (HFmrEF) (LVEF 41–49%) [3]. Acute and long-term follow-up prognostic outcomes differ in different subtypes and ethnicities [2].

Exercise intolerance is defined as an impaired capacity to perform physical activities accompanied by dyspnea and/or fatigue. Poor heart pumping and filling ability in HF leads to exercise intolerance. The mechanisms of exercise intolerance in HF are multifactorial, including impaired cardiac and pulmonary reserve and reduced skeletal muscle perfusion and function [4]. Since exercise intolerance causes reduced quality of life and increased mortality, evaluating patients’ exercise tolerance is important [4]. The following items help us to quantify the degree or severity of exercise intolerance that affect functional capacity: the New York Heart Association (NYHA) functional classification, quality of life assessment, electrocardiogram (ECG) stress testing, six-minute walking test (6MWT) and cardiopulmonary exercise testing (CPET).

CPET is the gold standard in assessing aerobic capacity and formulating function-based prognostic stratification. Among CPET parameters, oxygen uptake at peak exercise (peak VO_2_) and the slope of the relationship between minute ventilation and carbon dioxide production (V_E_/VCO_2_ slope) are most frequently used to assess HF severity, short- and long-term prognosis and the patient selection of heart transplantation [5]. In addition to these two parameters, other CPET variables including exercise oscillatory ventilation (EOV) and the partial pressure of end-tidal CO_2_ (P_ET_CO_2_) during rest and exercise also demonstrated strong prognostic value in HFrEF patients [6].

Acute decompensated heart failure is defined as newly onset or worsening symptoms and signs of HF. It may be related to structural or functional cardiac dysfunction resulting from acute coronary syndrome or left ventricular dysfunction; it eventually presents with pulmonary congestion and systemic congestion-related organ dysfunction [7]. Patients with acute decompensated heart failure after medical treatment usually remain with markedly reduced exercise capacity, leading to early termination when performing CPET. There are several criteria to confirm maximal effort during CPET, including VO_2_ and heart rate plateau with increased workload, rating of perceived exertion >17 on a 6–20 scale or >7 on a 0–10 scale and the most objective indicator, peak respiratory exchange ratio (RER) > 1.1. Peak RER < 1.1 is regarded as not reaching maximal effort during CPET [8]. For those who have difficulty providing maximal effort or satisfying objective criteria for a maximal exercise test, submaximal parameters play an important role. One of the submaximal parameters, oxygen uptake efficiency slope (OUES), recently has revealed high value in HF patients. The OUES is defined as the relationship between oxygen consumption (absolute $\dot{V}$O_2_ (mL·min^−1^)) and minute ventilation ($\dot{V}$_E_ (L·min^−^^1^)). This relationship represents how efficiently the musculoskeletal system extracts oxygen from the cardiopulmonary system during exercise. The advantages of OUES are the excellent test-retest reliability, high correlation with peak VO_2_ and relative stability during the incremental exercise test [9].

The 2021 European Society of Cardiology guidelines for the diagnosis and treatment of acute and chronic HF reveal the advantages of exercise training in HF patients, including the improvement in exercise tolerance, health-related quality of life and decreased rate of re-hospitalization [3]. Although the importance of cardiac rehabilitation cannot be overemphasized in chronic HF patients, little is known about the benefits of cardiac rehabilitation for acute HF patients under safety concerns [4]. Kaneko et al. reports several prognostic advantages after early initiation of phase I cardiac rehabilitation in acutely decompensated HF patients [10]. CPET can provide physiatrists with additional information on exercise tolerance and potential risks so as to recommend optimal exercise intensity for acute decompensated HF patients; however, few CPET are performed before discharge for safety concerns. In addition, the prognostic value of OUES in the pre-discharge status of patients with acute decompensated HF remains unclear. Hence, our current study aims to investigate the pre-discharge cardiorespiratory fitness of HFrEF patients after phase I cardiac rehabilitation and to determine the prognostic value of OUES as a submaximal CPET parameter in HFrEF patients.

## 2. Materials and Methods

### 2.1. Participants

We recruited patients of HFrEF with acute decompensation during hospitalization between September 2017 and June 2020 from one medical center. The inclusion criteria were patients older than 18 years of age, diagnosed with acute decompensated heart failure, and LVEF less than 40% on echocardiography. The exclusion criteria were patients too fragile for cardiopulmonary testing or training, including being bedridden longterm for more than 3 months, having cognitive impairment or neuromuscular disorders with unfavorable rehabilitation potential, being ventilator-dependent or having severe pulmonary disorder with oxygen dependency. The treatments for acute decompensated HF during hospitalization including medication adjustment and interventional procedures, if any, were carried out by cardiologists. Patients without immediate complications after treatments were consulted for phase I cardiac rehabilitation. The phase I cardiac rehabilitation training was performed according to Kaohsiung Veteran General Hospital cardiac rehabilitation training protocol, modified from the American College of Sports Medicine (ACSM) guidelines [8]. The training items included muscle strength, endurance training, long sitting, transferring and then progressive walking as tolerated, with a target heart rate of baseline heart rate plus 20 beats/min. All physiotherapists using this protocol had at least 3 years of experience in executing cardiac rehabilitation. The pre-discharge functional capacity assessments were evaluated by 6MWT and CPET, which were performed 1–2 days before discharge. This study was approved by the Institutional Review Board of Kaohsiung Veteran General Hospital (project number: VGHKS17-CT11-11), and patients agreed to informed consent before participation.

Patients were followed for one year after discharge, and medical care was continuously provided at the outpatient clinic of the Department of Cardiology. Major adverse cardiovascular events (MACE) during the one-year follow-up were identified from medical records and confirmed by cardiologists. In this study, MACE was defined as cardiovascular death, myocardial infarction, stroke, hospitalization related to heart failure and revascularization, including percutaneous coronary intervention and coronary artery bypass graft [11]. Patients with MACE were set as having an event.

### 2.2. Exercise Testing

The CPET was performed by using a MetaLyzer 3B (Cortex Biophysik GmbH Co., Leipzig, Germany) system including a leg ergometer, a gas analyzer and an ECG monitor to measure the exercise capacity of patients. The incremental workload of 10 W/min was performed with all CPET being performed by a physiatrist with more than 10 years of experience.

Direct measurements of oxygen consumption (VO_2_), carbon dioxide production (VCO_2_), minute ventilation (V_E_), respiratory rate and several derived variables such as RER and slope of VO_2_/VCO_2_ were obtained. The measurement of anaerobic threshold (AT) was commonly determined when the VCO_2_−VO_2_ slope abruptly increased, with OUES calculated by linear regression between VO_2_ and log (V_E_) with the equation: VO_2_ = a log (V_E_) + b. The slope “a” was determined as the OUES.

A 12-lead ECG monitor was continuously used during exercise testing. Subjects were exercised to their self-determined maximal capacity or until the physiatrist stopped the test.

### 2.3. Statistical Analysis

Student *t*-test was used to compare between-group differences in continuous variables with the chi-squared test comparing between-group differences in categorical variables and the Shapiro–Wilk test determining if continuous variables followed a normal distribution. Receiver operating characteristic (ROC) curves were plotted and the optimal threshold values for exercise capacity measures for predicting one-year MACE were determined by selecting the point at which the maximum summation value of sensitivity and specificity was achieved. Kaplan–Meier survival analysis and the log-rank test were used to analyze differences in MACE between groups with multivariate Cox regression analysis being used to estimate the hazard ratio (HR) of potential prognostic factors. All statistical analyses were performed by using SPSS version 19. A two-tailed *p*-value of less than 0.05 was considered to be statistically significant.

## 3. Results

A total of 85 patients were included, with 64 males and 21 females. There were 43 patients having MACE in one year, including 6 (14.0%) cardiac deaths, 21 (48.8%) re-hospitalizations for medical treatments related to acute HF, and 16 (37.2%) re-hospitalizations for coronary intervention or surgical interventions such as coronary artery bypass grafting or valve replacement. The basic demographics of all enrolled patients are shown in Table 1 with the median, 25th and 75th percentile values shown for continuous variables that were not distributed normally. All patients were classified as classes II and III according to NYHA classification. The demographics, clinical data and exercise capacity parameters between the HF patients with and without MACE in one year are listed in Table 2. Those without MACE had higher OUES (*p* = 0.010) than patients with MACE. Furthermore, the demographics, clinical data and exercise capacity parameters between RER ≥ 1.1 and RER < 1.1 are listed in Table 3. Patients with RER ≥ 1.1 had a younger age (*p* = 0.022), higher peak heart rate (*p* = 0.028), higher peak V_E_ (*p* = 0.001), higher chronotropic index (*p* = 0.006) and longer walking distance on 6MWT (*p* = 0.033).

**Figure 1:**
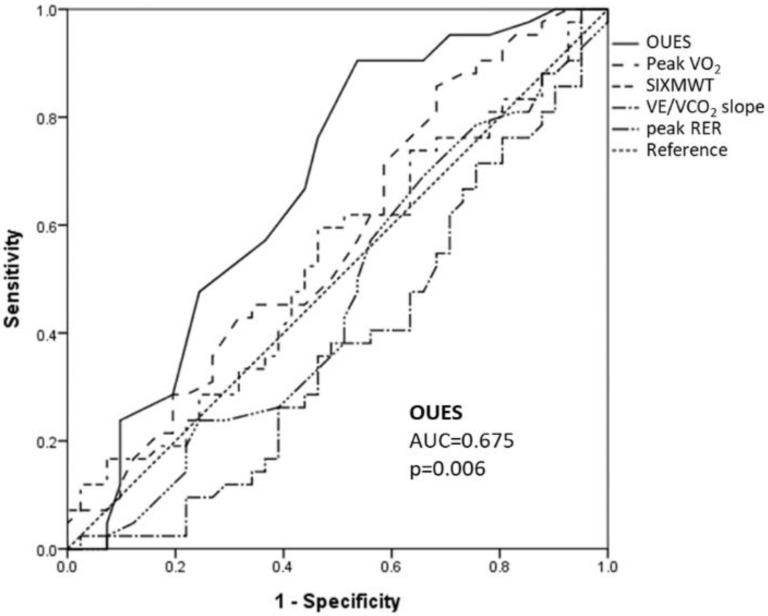
Receiver operating characteristic curves of exercise capacity measures in predicting one-year major adverse cardiovascular event (MACE). Oxygen uptake efficiency slope showed the highest area under the curve with statistical significance in predicting one-year MACE in heart failure patients.

For predicting one-year MACE, the ROC curves of OUES, peak VO_2_, V_E_/VCO_2_ slope, 6MWT and peak RER were analyzed. The area under curve (AUC) values listed in descending order included OUES 0.675 (*p* = 0.006), peak VO_2_ 0.572 (*p* = 0.259), 6MWT 0.531 (*p* = 0.626), peak RER 0.459 (*p* = 0.524) and VE/VCO_2_ slope 0.381 (*p* = 0.063), with only OUES reaching statistical significance (Figure 1). The optimal cut-off point in predicting one-year MACE of OUES was 1.25, which is determined by maximal summation of sensitivity and specificity.

**Figure 2:**
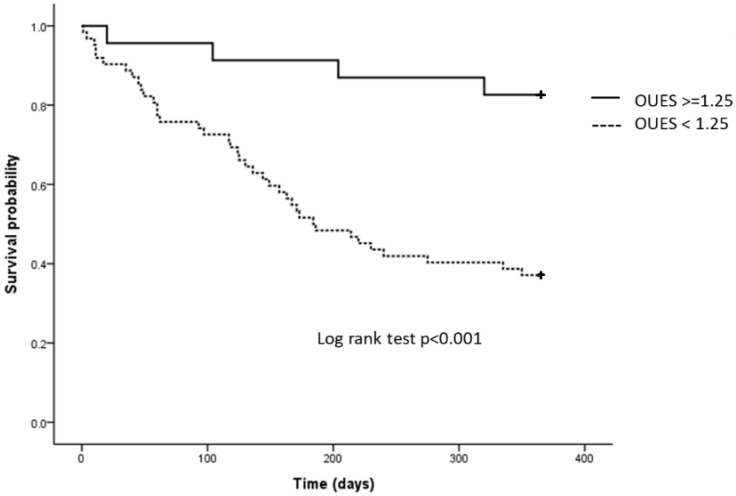
Kaplan–Meier analysis of one-year major adverse cardiovascular event (MACE) in heart failure patients with high and low oxygen uptake efficiency slopes (OUES). Heart failure patients with low OUES showed significantly higher rates of one-year MACE than those with high OUES (*p* < 0.001).

The one-year MACE in our study group was 49.4%. Kaplan–Meier analysis and the log-rank test revealed a statistically significant difference between the one-year MACE of the acute decompensated HF patients with high and low OUES (*p* < 0.001) (Figure 2). After adjusting for age, gender, NYHA, underlying disease and medications, further multivariate Cox regression analysis results are shown in Table 4. The multivariate Cox regression analysis showed patients with lower OUES had increased risk of one-year MACE, with a hazard ratio of 5.421 (*p* = 0.004). The post hoc sample size calculation for survival analysis was performed using an online calculator (powerandsamplesize.com, accessed on 7 September 2022) provided by HyLown Consulting (Atlanta, Georgia), formula for Cox proportional hazard model, setting type I error rate 0.05, power 0.8, hazard ratio 5.4, overall probability of event 0.5 and equivalence margin 0.5, yielding a sample size of 62. In comparison to other prognostic predictors listed in Table 4, including VO_2_, V_E_/CO_2_ and 6MWT, OUES was a significantly better predictor in predicting one-year MACE in HFrEF patients.

## 4. Discussion

Our current study suggests declined functional performance and low peak oxygen consumption (45.5% of predicted peak VO_2_) in acute decompensated HFrEF patients at pre-discharge status. In addition, OUES provided better one-year MACE prediction than other CPET parameters in this population.

There are several predictors other than blood tests for risk of mortality in patients with acute HF. A recent report suggested hydration status evaluated by bioimpedance vector analysis, along with brain natriuretic peptide (BNP), blood urea nitrogen and arterial blood gas generated reliable predictive value on long-term mortality risk in acute decompensated HF patients [12]. This emphasized the critical role of multiparametric approaches to provide comprehensive assessment of HF patients in clinical practice. In chronic HF patient, cardiac rehabilitation poses several advantages in cardiopulmonary fitness, including improved exercise capacity, autonomic function, endothelial function and less depressive symptoms and leads to left ventricular reverse remodeling [10]. According to Kaneko et al., the early initiation of phase I cardiac rehabilitation training in hospitalized patients with acute decompensated HF showed better short-term prognostic outcomes, including lower in-hospital mortality, shorter hospital stays and lower incidence of 30-day readmission rate [10]. However, there was limited information about the cardiorespiratory fitness condition in HF patients after acute decompensation. Few CPET are performed before discharge under safety concerns for acute the decompensated HFrEF population. Hence, this study emphasized the value of performing pre-discharge CPET on acute HFrEF patients. It not only provides information on current cardiorespiratory fitness under safe work and recreational load, but also poses an exercise prescription guide for the goal setting on phase II cardiac rehabilitation.

Decreased exercise capacity is a key symptom in HF patients [13]. CPET variables not only represent cardiorespiratory fitness, but also pose prognostic values. Among all the CPET parameters, peak VO_2_ and the V_E_/VCO_2_ slope are most frequently used and pose good prognostic value in chronic systolic HF populations [3,14]. Other parameters, including EOV and P_ET_CO_2_ during rest and exercise also demonstrated strong prognostic value in the same populations. According to Guazzi et al., the following items are poor prognostic indicators after 4-year follow-up in chronic HF populations: V_E_/VCO_2_ slope ≥45, peak VO_2_ < 10 mL/min/kg, presentation of EOV, resting P_ET_CO_2_ < 33 mmHg and exercising P_ET_CO_2_ < 3 mmHg [6].

Acute decompensated HF patients have the characteristic of being less likely to achieve the criteria of peak effort determined by ACSM guidelines. In our study population, only 47% achieved peak effort, which was determined by RER ≥ 1.1 at peak exercise stage. Patients who achieved peak effort during CPET had the characteristics of younger age, higher peak heart rate, higher peak V_E_, higher chronotropic index and longer distances on 6MWT. It is difficult to appropriately interpret the exercise capacity with peak VO_2_ in the acute decompensated HF population when only submaximal effort was achieved. Moreover, our results suggested no significant predictive value of one-year MACE with peak RER, in terms of ROC curve analysis (Figure 1). This suggested the important role of submaximal exercise parameters such as OUES in determining exercise capacity and the prognostic value for future cardiac events in acute decompensated HF patients.

The prognostic value of OUES in patients with chronic HF has received more attention lately, reporting a cut-off value of 1.4 in chronic HF [15] and 1.6 in end-stage HF [16]. However, there is a lack of suitable submaximal parameters for evaluation of the prognostic effect on the acute decompensated HF population. In the current study, we focused on an HFrEF acute decompensation population, the pre-discharge CPET was performed with MACE evaluated at one-year follow-up. We observed lower OUES provided better prognostic outcome prediction in one-year MACE than other prognostic predictors. The cutoff point of OUES is 1.25, with low OUES showing a 5.4-fold increased risk of one-year MACE over those with high OUES in our HFrEF acute decompensation population. In addition, we noticed no difference in OUES between those achieving peak effort or not, as shown in Table 3, which indicate that OUES is independent of RER reached during CPET in acute decompensated HF patients. This characteristic of OUES makes it a potentially more suitable prognostic marker for those HFrEF acute decompensation populations with less capability of achieving maximal effort.

OUES is also used as a cardiorespiratory fitness parameter in other diseases. Tsai et al. reported OUES as a valuable parameter to evaluate the exercise capacity of post-acute myocardial infarction patients after phase I cardiac rehabilitation; the post-training OUES poses stronger prognostic value than baseline OUES in coronary artery disease patients [17]. Buys et al. also reported lack of improvement in OUES after an exercise training program revealed worse prognostic outcomes [18]. In children with congenital heart disease, the OUES was significantly impaired vis-à-vis normal children [14]. In children with total repair of tetralogy of Fallot, OUES as normalized by body surface area and peak VO_2_ are useful predictors of two-year cardiac-related hospitalization [19]. The current evidence suggests the potential prognostic value of OUES in cardiovascular diseases, while also providing a guide to exercise prescription and determining outcome of cardiac rehabilitation training.

In addition to the NYHA functional classification and CPET results, 6MWT is a simple, inexpensive and well-tolerated test for whom a maximal exercise test cannot be obtained. Previous studies have shown relationship between these parameters. There are mild to moderate inverse correlations between NYHA classifications to 6MWT distances and V_E_/VCO_2_ slope to 6MWT distances, and moderate to strong correlations between peak VO_2_ to 6MWT distances. In addition to chronic HF, decreased 6MWT distances is one of the strongest independent predictors of long-term mortality and HF hospitalizations in acute HF patients [20,21]. Distance less than 300 m indicated poor prognosis, and distance less than 200 m indicated increased risk of death in chronic HF patients [20].

Grundtvig et al. reported the cut-off values of 380 m in 6MWT to predict all-cause mortality after 2-year follow-up in outpatient CHF populations [22]. Chen et al. also reported a cut-off value of 330 m in 6MWT to predict 2-year mortality in patients with pulmonary arterial hypertension [23]. According to this previous research, we chose 330 m as the prognostic cut-off value in acute decompensated HF patients. However, current results did not observe significant prognostic prediction of 6MWT in one-year MACE in acute decompensated HFrEF patients.

There are several limitations to this study. First, the study population was relatively small and could only refer to HFrEF populations. One of the reasons for the small sample size was related to some restrictions when enrolling patients with acute decompensated heart failure for early CPET before discharge. Though the study group was small, a post hoc power calculation was performed yielding a power of 0.97. Further investigation should be carried out in large-scale studies and should also include HFpEF and HFmrEF populations. Additionally, patients were recruited from a veteran’s hospital, so the majority of our patients were male, and, furthermore, CPET data was lacking at the one-year follow-up, which might have provided further validation on value of OUES as a prognostic marker in the acute decompensated HF population.

## 5. Conclusions

The current results suggest OUES as a significant prognostic parameter in acute HF patients at one-year follow-up, while further emphasizing the critical role of CPET in patients with acute HF populations for prognostic stratification and guidance for exercise interventions in executing cardiac rehabilitation programs.

## Figures and Tables

**Table 1:** Basic demographics of all enrolled patients.

	All Patients (*n* = 85)				
	*n* (%)	Mean ± SD	25th	Median	75th
Age (year)		61.33 ± 14.30			
Gender					
Male	64 (75.3)				
Female	21 (24.7)				
Height (cm)		163.93 ± 10.94	156.40	165.30	170.60
Weight (kg)		69.13 ± 19.10	58.30	63.90	75.20
BMI (kg/m^2^)		25.22 ± 5.93	22.05	24.20	26.89
NYHA classification					
II	23 (27.1)				
III	62 (72.9)				
HF etiology					
DCM	12 (14.1)				
MR	23 (27.1)				
CAD	35 (41.2)				
Obesity	1 (1.2)				
AS	4 (4.7)				
Thrombus	2 (2.4)				
CTD	1 (1.2)				
Unknown	7 (8.2)				
Comorbidities					
CVA	4 (4.7)				
Hypertension	63 (74.1)				
DM	31 (36.5)				
Dyslipidemia	31 (36.5)				
PAOD	5 (5.9)				
ESRD	3 (3.5)				
Medications					
ACEI/ARB/ARNI	74 (87.1)				
Beta-blockers	69 (81.2)				
Diuretics	68 (80.0)				
MRA	56 (65.9)				
Direct vasodilators	9 (10.6)				
Digitalis	6 (7.1)				
Sinus node inhibitor	40 (47.1)				
LVEF (%)		30.52 ± 7.42			
BNP (pg/mL)		1390.29 ± 1395.39 (*n* = 75)	324.00	884.10	2209.00
ATVO_2_ (mL/min/kg)		7.72 ± 2.45	5.95	7.35	8.93
AT heart rate		91 ± 15	79	89	102.5
PeakVO_2_ (mL/min/kg)		10.85 ± 3.49			
Percent predict peakVO_2_		45.55 ± 16.72	34.34	45.41	54.49
Peak heart rate		103 ± 20	90	101	117.5
6MWT (m)		260.97 ± 113.66 (*n* = 83)	192.0	276.0	343.2
HR after 6MWT		87 ± 14	76	86	100
Peak VE (L/min)		33.73 ± 12.46	25.27	32.00	40.85
HRR		8 ± 6	4	7	13
VE/VCO_2_ slope		40.73 ± 11.12	34.05	40.30	45.10
OUES		1.03 ± 0.41			
ECP		5.89 ± 5.40	4.10	5.06	6.46
VO_2_/WR slope		7.85 ± 4.54	5.55	7.70	9.40
Chronotropic index		0.34 ± 0.21	0.20	0.32	0.46

ACEI = angiotensin-converting enzyme inhibitor; ARB = angiotensin II receptor blocker; ARNI = angiotensin receptor-neprilysin inhibitor; AS = aortic stenosis; BMI = Body Mass Index; CAD = coronary artery disease; CTD = connective tissue disease; CVA = cerebrovascular accident; DCM = dilated cardiomyopathy; DM = diabetes mellitus; ESRD = end stage renal disease; HF = heart failure; MR = mitral regurgitation; MRA = mineralo-cortocoid receptor antagonist; NYHA = New York Heart Association; PAOD = peripheral arterial occlusion disease; SD = standard deviation. LVEF = left ventricular ejection fraction; BNP = B-type natriuretic peptide; AT = anaerobic threshold; VO_2_ = oxygen uptake; 6MWT = six-minute walking test; VE = minute ventilation; HRR = heart rate reserve; VCO_2_ = volume of exhaled carbon dioxide; OUES = oxygen uptake efficiency slope; ECP = exercise cardiac power; WR = work rate; SD = standard deviation.

**Table 2:** Basic demographics and exercise capacity measures of heart failure patients with and without major adverse cardiovascular events at one-year follow-up.

	No MACE (*n* = 42)		MACE (*n* = 43)		*p*-Value
	*n*	Mean ± SD	*n*	Mean ± SD	
Age (year)		58.50 ± 14.30		64.09 ± 13.91	0.071
Gender					0.850
Male	32		32		
Female	10		11		
Height (cm)		164.40 ± 12.14		163.47 ± 19.75	0.698
Weight (kg)		73.83 ± 22.48		64.55 ± 13.88	0.026
BMI (kg/m^2^)		26.37 ± 7.16		24.11 ± 4.22	0.079
NYHA classification					0.385
II	14		9		
III	28		34		
LVEF (%)		30.48 ± 7.51		30.56 ± 7.41	0.960
BNP (pg/mL)		1183.11 ± 1226.85 (*n* = 39)		1614.74 ± 1543.67 (*n* = 36)	0.183
ATVO_2_ (mL/min/kg)		8.23 ± 2.79		7.22 ± 1.98	0.056
AT heart rate		92 ± 18		90 ± 12	0.629
PeakVO_2_ (mL/min/kg)		11.38 ± 3.86		10.33 ± 3.05	0.169
Peak heart rate		102 ± 22		104 ± 18	0.742
6MWT (m)		268.47 ± 107.78 (*n* = 41)		253.65 ± 119.97 (*n* = 42)	0.556
HR after 6MWT		86 ± 14		88 ± 14	0.502
Peak VE (L/min)		35.02 ± 13.56		32.47 ± 11.30	0.347
HRR		8 ± 5		9 ± 7	0.432
V_E_/VCO_2_ slope		39.21 ± 11.96		42.22 ± 10.16	0.214
OUES		1.15 ± 0.47		0.92 ± 0.31	0.010
ECP		5.77 ± 2.41		6.01 ± 7.26	0.844
VO_2_/WR slope		7.79 ± 3.11		7.91 ± 5.64	0.905
Chronotropic index		0.32 ± 0.22		0.36 ± 0.20	0.302

AT = anaerobic threshold; BNP = B-type natriuretic peptide; ECP = exercise cardiac power; HR = heart rate; HRR = heart rate reserve; LVEF = left ventricular ejection fraction; MACE = major cardiac event; NYHA = New York Heart Association; OUES = oxygen uptake efficiency slope; SD = standard deviation; VCO_2_ = volume of exhaled carbon dioxide; V_E_ = minute ventilation; VO_2_ = oxygen uptake; WR = work rate; 6MWT = six-minute walking test.

**Table 3:** Demographics and exercise capacity measures of heart failure patients with and without achieving maximal effort during exercise testing.

	RER < 1.1 (*n* = 45)		RER ≥ 1.1 (*n* = 40)		*p*-Value
	*n*	Mean ± SD	*n*	Mean ± SD	
Age (year)		64.64 ± 15.61		57.60 ± 11.76	0.022
Gender					0.077
Male	30		34		
Female	15		6		
Height (cm)		161.92 ± 12.66		166.20 ± 8.19	0.072
Weight (kg)		67.24 ± 16.53		71.26 ± 21.65	0.335
BMI (kg/m^2^)		24.88 ± 4.31		25.61 ± 7.39	0.577
NYHA classification					0.485
II	12		11		
III	33		29		
LVEF (%)		31.36 ± 7.78		29.58 ± 6.95	0.272
BNP (pg/mL)		1409.62 ± 1504.40 (*n* = 41)		1366.99 ± 1273.55 (*n* = 34)	0.896
ATVO_2_ (mL/min/kg)		7.58 ± 2.59		7.88 ± 2.31	0.587
AT heart rate		89 ± 14		94 ± 17	0.174
PeakVO_2_ (mL/min/kg)		10.21 ± 3.34		11.56 ± 3.56	0.076
Peak heart rate		99 ± 16		109 ± 24	0.028
6MWT (m)		235.39 ± 114.99 (*n* = 43)		288.47 ± 106.89 (*n* = 40)	0.033
HR after 6MWT		89 ± 15		87 ± 15	0.556
Peak VE (L/min)		29.48 ± 9.30		38.51 ± 13.87	0.001
HRR		8 ± 6		9 ± 7	0.463
V_E_/VCO_2_ slope		42.38 ± 12.81		38.87 ± 8.64	0.147
OUES		0.99 ± 0.37		1.07 ± 0.45	0.364
ECP		5.04 ± 1.81		6.85 ± 7.57	0.123
VO_2_/WR slope		7.92 ± 5.80		7.78 ± 2.53	0.894
Chronotropic index		0.28 ± 0.17		0.41 ± 0.23	0.006

AT = anaerobic threshold; BNP = B-type natriuretic peptide; ECP = exercise cardiac power; HR = heart rate; HRR = heart rate reserve; LVEF = left ventricular ejection fraction; NYHA = New York Heart Association; OUES = oxygen uptake efficiency slope; RER = respiratory exchange ratio; VCO_2_ = volume of exhaled carbon dioxide; VE = minute ventilation; VO_2_ = oxygen uptake; WR = work rate; SD = standard deviation; 6MWT = six-minute walking test.

**Table 4:** Predictive measures for one-year major adverse cardiovascular event in patients with acute decompensated heart failure.

Variable	No. of Patients	MACE	HR ^†^	95% CI
OUES				
>1.25	23	4	1.00	
<1.25	62	39	5.421 **	1.694 to 17.347
Peak VO_2_				
>10 mL/min/kg	50	23	1.00	
<10 mL/min/kg	35	20	1.208	0.574 to 2.544
V_E_/VCO_2_ slope				
<45.0	62	31	1.00	
>45.0	23	12	0.962	0.424 to 2.184
6MWT				
>330 m	24	11	1.00	
<330 m	61	32	0.583	0.249 to 1.368

** *p* < 0.01. ^†^ adjusted for age, gender, NYHA, underlying diseases, and medications. CI = confidence interval; HR = Hazard Ratio; MACE = major cardiac event; OUES = oxygen uptake efficiency slope; VCO_2_ = volume of exhaled carbon dioxide; VE: minute ventilation; VO_2_ = oxygen uptake; 6MWT = six-minute walking test.

## Data Availability

Not applicable.
